# Optimizing human coronavirus OC43 growth and titration

**DOI:** 10.7717/peerj.13721

**Published:** 2022-07-08

**Authors:** Christopher Savoie, Roger Lippé

**Affiliations:** 1 Centre de Recherche du CHU-Sainte-Justine, Montreal, Quebec, Canada; 2 Department of Pathology and Cell biology, University of Montreal, Montreal, Quebec, Canada

**Keywords:** HCoV-OC43, OC43, SARS-CoV-2, Propagation, Titration, Viral stocks, COVID

## Abstract

Coronaviruses have been at the forefront of the news for the last 2 years. Unfortunately, SARS-CoV-2, the etiologic agent for the COVID-19 pandemic, must be manipulated in biosecurity level 3 settings, which significantly limits research. Meanwhile, several less pathogenic human coronaviruses (HCoV) exist and can be studied in much more common biosafety level 2 laboratories. Among them, HCoV-OC43 is a good surrogate candidate for SARS-CoV-2 since both are phylogenetically related human *Betacoronaviruses*. However, one issue has been the lack of standardized means among laboratories to propagate and titer this less virulent coronavirus. The present study probes the optimal parameters to propagate HCoV-OC43. First, testing of five different cell lines (MRC-5, Huh7.5, Vero, HCT-8, HRT-18) indicated that the physiologically relevant MRC-5 human lung cell line produced among the highest viral titers. HRT-18 may however be an interesting alternative as they are quick growing cells that also led to higher viral titers and a better tropism for various HCoV-OC43 variants. We also probed the impact of serum and temperature during viral expansion and confirmed that the normal temperature of the upper respiratory track (33 °C) improves viral yields over the typical 37 °C used to grow many other viruses. Meanwhile, we did not notice any evidence that serum concentrations significantly affected the virus but interestingly noted that the virus grew quite efficiently in a serum-free media formulation. Meanwhile sonication of viral stocks somewhat improved viral titers. Four titration methods (plaque assays, TCID_50_-CPE, TCID_50_-IFA and TCID_50_–IPA) were also probed using two cell lines (VeroE6 and HRT-18). In our hands, plaque assays proved unreliable and quantification of the virus by scoring CPE positive wells was significantly less sensitive than antibody-based assays (IFA and IPA). While the latter methods were equally sensitive, we favor the TCID_50_-IPA method since simpler, faster and cheaper than the IFA protocol. Moreover, the HRT-18 cells appeared more sensitive to quantify the virus. Perhaps most importantly, these optimized protocols routinely led to high titer viral stocks in the order of 10^8^ TCID_50_/ml magnitude, which should fulfill the requirements of most experimental settings.

## Introduction

The COVID-19 pandemic reminds us of the importance of developing tools and methods to study coronaviruses. Among them are the endemic human coronaviruses HCoV-229E, -NL63, -OC43 and -HKU1 that have long been associated with mild respiratory diseases but also neurodegenerative diseases (Brison et al., 2014; Butler et al., 2006; Hicks et al., 2021). These belong to the same family as the more pathogenic and more recently characterized MERS-CoV, SARS-CoV and SARS-CoV-2, with which they share a similar viral life cycle. Unfortunately, studying these clinically critical coronaviruses require biosafety level 3 facilities, which is limiting. Meanwhile other human coronaviruses can be manipulated in widely available biosafety level 2 laboratories. Most interestingly, HCoV-OC43 is phylogenetically highly related to SARS-CoV-2 and belong to the same *Betacoronavirus* genera (Lu et al., 2020). It has thus been considered an appropriate surrogate virus for SARS-CoV-2 (Hu, Ma & Wang, 2022; Le Coupanec et al., 2021; Schirtzinger, Kim & Davis, 2021).

Despite years of research, HCoV-OC43 remains a somewhat difficult virus to grow so that optimized growth conditions and standardized titration methods are still lacking. Consequently, diverse cell lines have been tested including, but not limited to, FT (diploid fetal tonsil; (Schmidt, Cooney & Kenny, 1979), RD (heteroploid rhabdomyosarcoma; (Bracci et al., 2020; Hu, Ma & Wang, 2022; Schmidt, Cooney & Kenny, 1979), BS-C-1 (monkey kidney (Hu, Ma & Wang, 2022)), HCT-8 and HRT-18 (both human adenocarcinoma; (Hirose et al., 2021; Lambert et al., 2008c; Schirtzinger, Kim & Davis, 2021), MRC-5 (Bracci et al., 2020; Hu, Ma & Wang, 2022; Schirtzinger, Kim & Davis, 2021), Mv1Lu (mink lung epithelia; (Bracci et al., 2020)), Vero (Hirose et al., 2021) and VeroE6 (Hirose et al., 2021; Schirtzinger, Kim & Davis, 2021). Those studies also explored the optimal temperature to grow HCoV-OC43 and the impact of adding 1% to 8% of serum to the media during viral propagation. They also probed various titration methods including plaque assays using various overlays (agarose, avicel, methylcellulose) or the median tissue culture infectious dose (TCID_50_) based on the cytopathic effects (TCID_50_-CPE), immunofluorescence assays (TCID_50_-IFA) or immunoperoxydase assays (TCID_50_-IPA). Unfortunately, given the lack of recognizable cytopathic effects in many cell lines, it can be difficult to visually monitor the progression of the infection and quantify the virus by plaque assays (Hirose et al., 2021; Lambert et al., 2008c; Schmidt, Cooney & Kenny, 1979). The present work seeks to compare and optimize the current protocols and define the best and most sensitive means to detect HCoV-OC43. Our finding indicates that MRC-5 and HRT-18 cells are among the best cells to produce the virus and confirm that the cells produce more virus at 33 °C than 37 °C. Interestingly, the data revealed that it is possible to produce HCoV-OC43 in serum-free conditions as efficiently as in media containing up to 10% serum, which may be an advantage for some applications. Comparing four titration methods on two cell lines, we finally show that an indirect immunoperoxidase based TCID_50_ assay (TCID_50_-IPA) is the most practical and sensitive assay to monitor the virus and that the HRT-18 cells are better suited than the widely used VeroE6 cell line for titrations of coronaviruses. Most importantly, these optimized conditions routinely led to high titer viral stocks in the 10^8^ TCID_50_/ml order of magnitude.

## Materials and Methods

### Cell lines

MRC-5 human male lung cells (ATCC CCL-171) were maintained in Eagle’s Minimum Essential Medium (EMEM) (Wisent, Saint-Jean-Baptiste, QC, Canada) complemented with 10% HI-FBS, 1X non-essential amino acids (Gibco, Waltham, MA, USA), 1 mM sodium pyruvate (Wisent, Saint-Jean-Baptiste, QC, Canada) and 100 U/mL Penicillin & 100 μg/mL Streptomycin (Milipore Sigma, Burlington, MA, USA) (1X P/S). The HCT-8 human male ileocecal adenocarcinoma cell line (ATCC CCL-244) was maintained in Roswell Park Memorial Institute 1640 (RPMI-1640) with 10% heat inactivated fetal bovine serum (HI-FBS), 1X L-Glu and 1X P/S. The HRT-18 male human ileocecal adenocarcinoma (Gift from Dr. Talbot, INRS, QC, Canada (Mounir & Talbot, 1992) (distinct from ATCC CCL-244 which is listed as HCT-8/HRT-18) and Huh7.5 male human hepatoma (Gift from Dr. Soudeyns, CRCHUSJ) cell lines were maintained in Dulbecco’s Modified Eagle Medium (DMEM) (Wisent, Saint-Jean-Baptiste, QC, Canada) completed with 10% HI-FBS (Wisent, Saint-Jean-Baptiste, QC, Canada), 2 mM L-Glutamine (Milipore Sigma, Burlington, MA, USA) (1X) and 1X P/S. African green monkey kidney Vero (ATCC CCL-81) and VeroE6 (Gift from Dr. Liang, McGill University) female cell lines were maintained in DMEM containing 5% heat-inactivated (HI) bovine growth serum (BGS) (Wisent, Saint-Jean-Baptiste, QC, Canada), 1X L–Glu and 1X P/S.

### Viral strains and stocks

The original *Betacoronavirus* 1 HCoV-OC43 VR-759 and the HCoV-OC43 rOC/US183-2 double S protein mutant (H183R & Y241H) derived from VR-759 (herein described as VR-759 dm; gifts from Dr. Talbot, INRS, QC, Canada) were passaged twice on HRT-18 cells and harvested as previously described (Favreau et al., 2009). The HCoV-OC43 variant VR-1558 (ATCC, Manassas, VA, USA) was passaged twice on MRC-5 cells to obtain a working P3 stock. P3 viral stocks were independently prepared from the supernatant and the cell-associated fractions. Hence, cells were scraped and centrifuged at low speed (500×*g*, 10 min, 4 °C) to separate extracellular and cell-associated viruses. Extracellular virions were next concentrated by ultracentrifugation (60,000×*g*, 1 h, 4 °C) and resuspended in a minimal volume of DMEM while the intracellular viruses were released by two rounds of freeze-thawing. Both fractions were sonicated 15 times for 1s at a power of 8 of a Sonic Dismembrator Model 100 using a cup-horn setting (Fischer Scientific, Hampton, NH, USA), flash frozen in liquid nitrogen and stored at −80 °C. These stocks were then used to infect cells as described below.

### Viral infections

Unless otherwise specified, infections were carried on cells seeded in six-well plates grown to 80% confluence in maintenance media. The media was removed and a viral dilution of 500 µL per well, corresponding to a MOI of 0.7 (TCID_50_-IPA method), was added. Plates were incubated at 37 °C, 5% CO_2_ on a shaker for 1 h and 2 mL of infection media (DMEM 2% FBS, 1X L-Glu, 1X P/S) was added per well and the plate incubated at 33 °C, 5% CO_2_ for 3 days. The viruses released into the supernatant were then harvested and quantified as is, *i.e*. without ultracentrifugation.

### Virus titration by TCID_50_

Two hundred thousand VeroE6 or fifty thousand HRT-18 cells were seeded per well in 96-well plates and incubated at 37 °C, 5% CO_2_ until 80% confluent (1 or 3 days after seeding, respectively), leading to similar confluencies on the day of the infection. The media was removed and cells inoculated with 50 µL of a serial 10-fold dilution of samples (4 to 6 replicates) and incubated at 37 °C, 5% CO_2_ on a shaker for 1 h for viral adsorption. To each well, 50 µL of infection media (DMEM 2% FBS, 1X L-Glu, 1X P/S) was added and plates incubated at 33 °C, 5% CO_2_ for 4 days. For cytopathogenic effect (CPE), plates were read on an Evos XL Core microscope with a 20X objective (Invitrogen, Waltham, MA, USA). Thereafter, immunoperoxidase staining and revelation with 3,3′Diaminobenzidine (DAB) (TCID_50_-IPA method) or immunofluorescence staining (TCID_50_-IFA method) was performed as previously described but without washing the cells in 1X PBS prior to fixation (Lambert et al., 2008a; Schirtzinger, Kim & Davis, 2021). The primary antibody used for these staining methods was the 4.3E4 hybridoma antibody against the HCoV-OC43 S protein (Gift from Dr. Talbot, INRS, QC, Canada) diluted 1:50 in 1X PBS and the secondary antibodies were respectively the peroxidase-conjugated AffiniPure Goat Anti-Mouse IgG (H+L) (Jackson Laboratory, Bar Harbor, ME, USA) diluted 1:2000 in 1X PBS and the Chicken anti-Mouse IgG (H+L) Cross-Adsorbed Secondary Antibody Alexa Fluor 488 (Invitrogen, Waltham, MA, USA) diluted 1:40 in 1X PBS. A fresh stock of DAB was prepared by dissolving the pellets in Milli-Q water with HCl then diluted in 1X PBS to a final concentration of 30–40 mg/100 mL. DAB-stained plates were read after 15 min of staining as described above for CPE. Fluorescent-stained plates were read on a Leica DMi8 fluorescence microscope. The median tissue culture infectious dose (TCID_50_) is defined as the dilution of the virus necessary to infect 50% of cell cultures. It was calculated according to the Spearman & Karber method (Lei et al., 2021; Ramakrishnan, 2016) with the following formula: TCID_50_ = log (highest dilution giving 100% positive wells) + 0.5 − ((total number of positive wells)/(number of wells per dilution)). These values differ from the plaque forming units (PFU), but it can be mathematically estimated from the Poisson distribution by multiplying the TCID_50_ by 0.7 (Hierholzer & Killington, 1996).

### Flow cytometry

Infected MRC-5 cells were harvested 3 days post-infection (dpi) and fixed/permeabilized with the BD Cytofix/Cytoperm™ kit using undiluted 4.3E4 primary antibody (provided by Dr Pierre Talbot) and 1:40 diluted Chicken anti-Mouse IgG (H+L) Cross-Adsorbed Secondary Antibody Alexa Fluor 488 (Molecular Probes, Eugene, Oregon, USA). Data was collected using a BD FACS Canto II and analyzed with FlowJo™ 10.6.1.

### Statistics

All statistics were done on GraphPad Prism 9. One-way ANOVAs with Dunnett multiple comparisons were used to compare multiple conditions. Student’s t-tests were used to analyze other data. The difference between the experimental conditions were considered statistically significant when the *p*-value was less than 0.05.

## Results

### The human lung cell line MRC-5 is a good model to propagate HCoV-OC43

**Figure 1:**
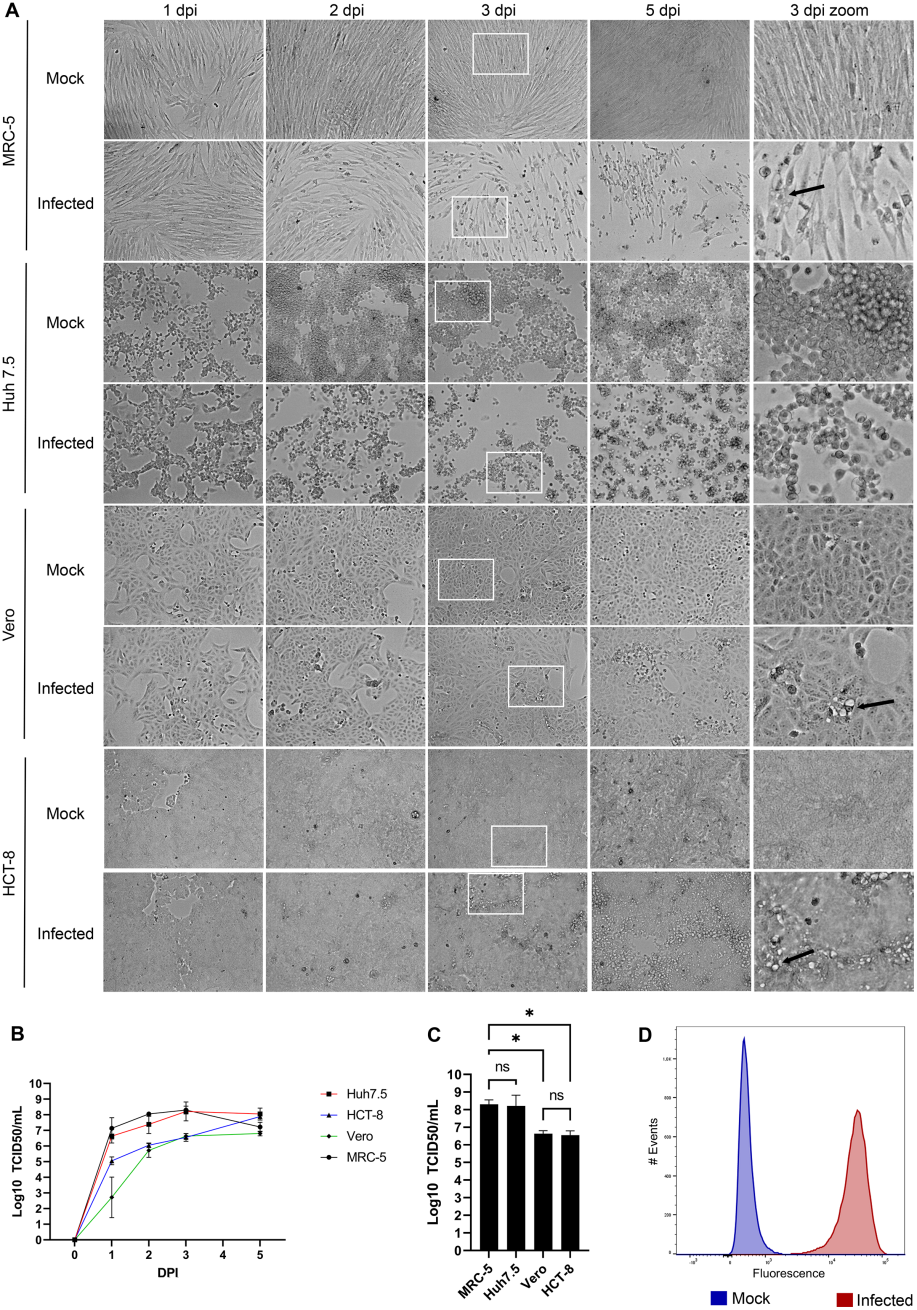
HCoV-OC43 grows best on the MRC-5 and Huh7.5 cell lines. For all panels, MRC-5, Huh7.5, Vero and HCT-8 cells were grown to 80% confluence on six-well plates then mock treated or infected with at an MOI of 0.7. The cells were then incubated for up to 5 days at 33 °C in DMEM containing 2% serum. (A) CPE was monitored by bright field microscopy at the indicated days post-infection (dpi). The right panels show a zoom view of CPE observed at 3 dpi. (B) To quantify the kinetics of propagation of HCoV-OC43, the extracellular virus produced by infected MRC-5 cells was quantified on HRT-18 cells using the TCID_50_ immunoperoxidase assay (TCID_50_-IPA) of Talbot and colleagues (see Materials and Methods). (C) MRC-5 cells infected for 3 dpi were fixed, permeabilized and HCoV-OC43 positive cells scored using viral specific antibodies (see Materials and Methods). (D) To compare viral yield among the cell lines, the extracellular virus harvested at 3 dpi was titered as above. Error bars represent SEM (*n* = 3). The titers were calculated with the Spearman and Karber method. Statistical analyses were done by one-way ANOVA with Dunnett multiple comparisons (*p < 0.05).

HCoV-OC43 is a slow growing virus that requires several days to reach its peak (Hirose et al., 2021; Hu, Ma & Wang, 2022; Lambert et al., 2008c; Schirtzinger, Kim & Davis, 2021). To first evaluate what might constitute a good host to propagate this virus, four susceptible cell lines were infected and examined over the course of several days. This included the African green monkey Vero cell line used to titer many viruses (Baer & Kehn-Hall, 2014), the HCT-8 human ileocecal adenocarcinoma cell line recommended by ATCC to grow HCoV-OC43, the physiologically more relevant MRC-5 human lung cell line (Bracci et al., 2020; Hu, Ma & Wang, 2022; Schirtzinger, Kim & Davis, 2021) and the Huh7.5 human hepatoma cell line given their high susceptible to the related SARS-CoV-2 and the impact of that virus on the liver (Chu et al., 2020; Mashraqi et al., 2022). As control, cells were mock infected to monitor their state over that same time span. Figure 1A shows that not all cell lines exhibited CPE. For instance, Vero and HCT-8 cells exhibited limited signs of infection up to 5 days post-infection (dpi). In contrast, both MRC-5 and Huh7.5 cells had significantly reduced confluency along with increased cell rounding, vacuolization and shrinking by 3 dpi, which subsisted but did not increase by day 5. Quantification of virions released in the supernatant by the TCID_50_-IPA method showed that viral titers peaked on the third day for all but the HCT-18 cell line, which required two more days to reach similar levels (Fig. 1B). Interestingly, MRC-5 and Huh7.5 cells produced nearly two more logs of virus on the third day (2.0 × 10^8^ and 1.7 × 10^8^ TCID_50_/ml respectively) than that obtained with Vero and HCT-8 cells (4.3 × 10^6^ and 3.6 × 10^6^ TCID_50_/ml respectively; Fig. 1C). Given the higher relevance of the MRC-5 (lung rather than liver cells) and their prevalence in the literature to grow, study and even isolate coronaviruses (Larson, Reed & Tyrrell, 1980; Min et al., 2020; Phillpotts, 1983), we opted to continue most of our experiments with the MRC-5 human lung cells, unless otherwise indicated. Moreover, MRC-5 infected cells were highly susceptible to HCoV-OC43, as determined by FACS using an antibody against HCoV-OC43, which indicated that 99% of the cells score positive for the virus (Fig. 1D).

### Optimal growth at 33 °C

**Figure 2:**
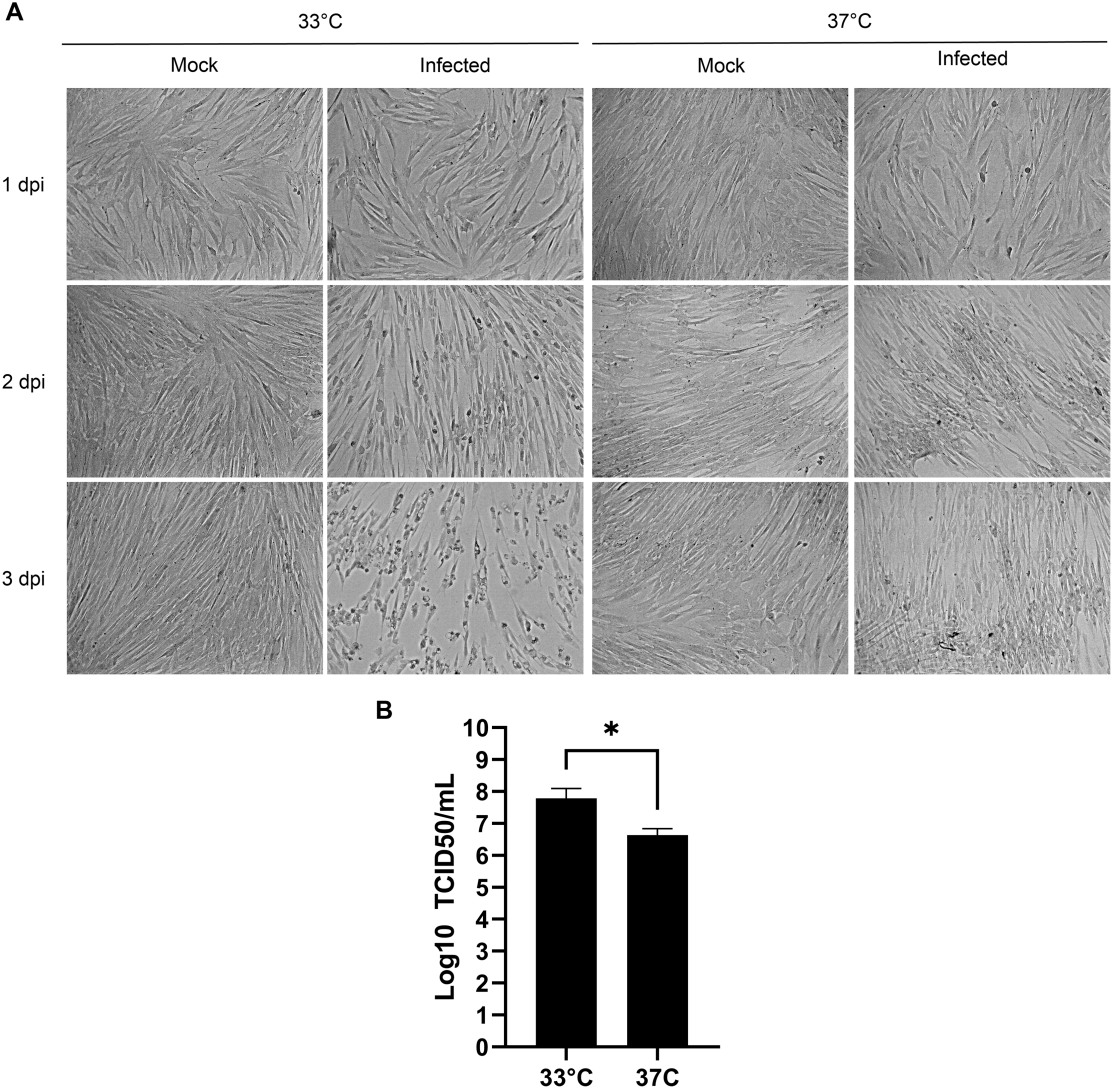
Optimal propagation of HCoV-OC43 at 33 °C. MRC-5 cells were grown to 80% confluence on 6-well plates before being mock treated or infected at an MOI of 0.7 and grown for 3 days at either 33 °C or 37 °C in DMEM supplemented with 2% serum. (A) Monitoring of the cells by bright field microscopy over time. Note that the infection leads to more CPE at 33 °C compared to 37 °C. (B) Extracellular virus harvested at 3 dpi were titered by the TCID_50_-IPA method as in Fig. 1. Error bars represent SEM (*n* = 3). Statistical analyses were done with a Student t-test (*p < 0.05).

Human viruses typically grow best at the cell body temperature of 37 °C. However, several publications have reported that coronaviruses and other respiratory viruses better propagate at 33 °C, which matches the temperature of the upper respiratory track (Bracci et al., 2020; Lamarre & Talbot, 1989; McFadden et al., 1985; V’kovski et al., 2021). To confirm the importance of temperature, MRC-5 cells were therefore infected with HCoV-OC43 and incubated for 3 days at either 33 °C or 37 °C. Upon examination by bright field microscopy, CPE was readily apparent at the lower temperature but absent at the higher temperature (Fig. 2A). Quantification of the viral yields in the tissue culture supernatant (Fig. 2B) confirmed this observation and were over a log greater at 33 °C (6.6 × 10^7^ TCID_50_/ml) than 37 °C (4.3 × 10^6^ TCID_50_/ml), which was statistically significant (*p* = 0.027). This indicated that HCoV-OC43 has adapted to the temperature of the upper respiratory tract and propagates better at 33 °C.

### Serum levels during the propagation does not alter viral yields

**Figure 3:**
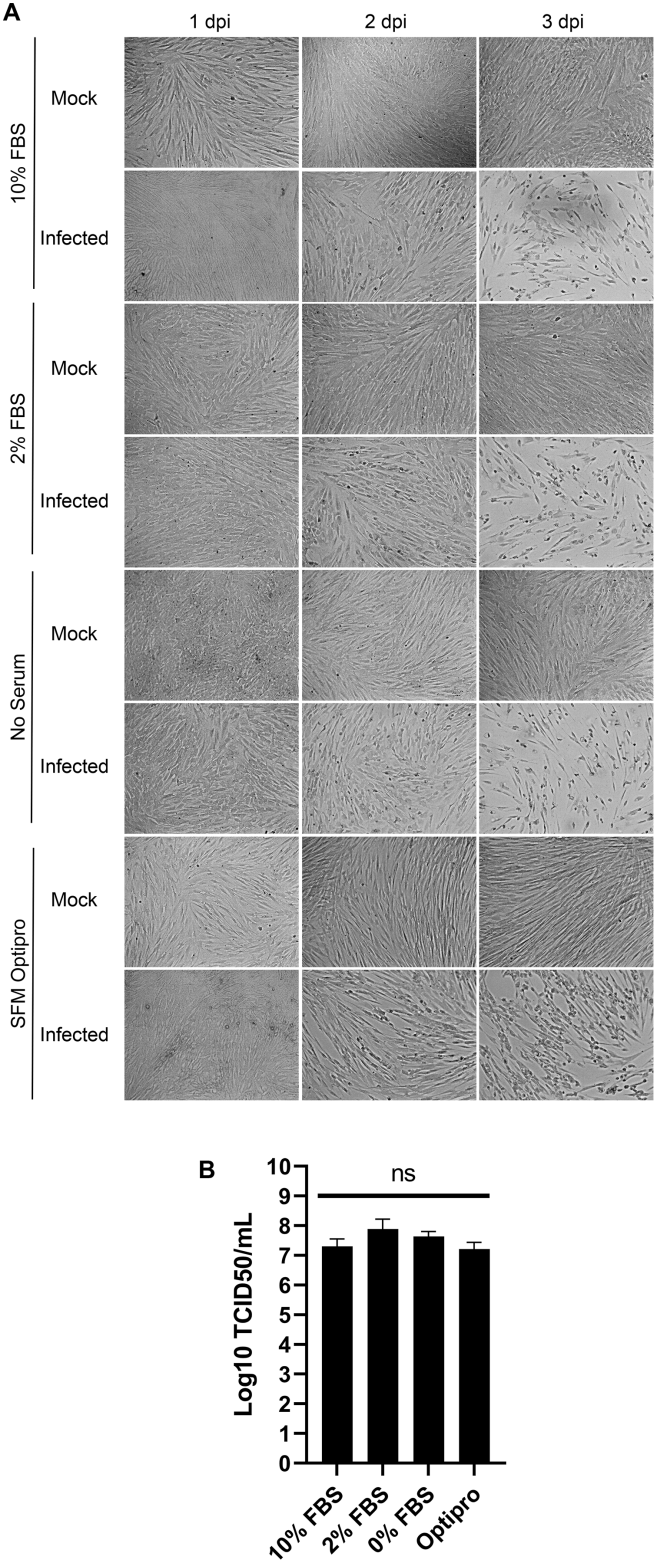
HCoV-OC43 can be grown in serum free media. MRC-5 cells were grown to 80% confluence on six-well plates then mock treated or infected at an MOI of 0.7 in serum free media. After a 1h adsorption period, cells were incubated for up to 3 days at 33 °C in the presence of EMEM containing different concentrations of serum (0%, 2% or 10%) or Optipro, a serum-free media (SFM-Optipro). (A) Bright field monitoring over the course of the infection indicated the lack of noticeable effect of the serum. (B) Extracellular virus harvested on at 3 dpi were titered by the TCID_50_-IPA method. Error bars represent SEM. Statistical analyses were done by one-way ANOVA with Dunnett multiple comparisons (*n* = 3).

In the literature, serum is used at concentrations in the tissue culture media from 1% to 8% (Bracci et al., 2020; Lambert et al., 2008b). To probe whether this is a critical parameter, MRC-5 cells were mock treated or infected with HCoV-OC43 and incubated for 3 days in DMEM complemented with decreasing concentrations of serum (10%, 2% and 0%) or in SFM-Optipro, a commercial serum-free formulation adapted to tissue culture. Daily inspection of the uninfected cells by bright-field microscopy revealed that they remained healthy over the 3 days of the experiments even without serum (Fig. 3A). Using the SFM-Optipro also yielded equally healthy and abundant cells, though they grew slightly slower and required passaging at lower dilutions. Albeit 2% serum was consistently slightly better, no statistically significant effect was noted on viral yields with titers ranging between 1.7 × 10^7^ and 7.7 × 10^7^ TCID_50_/ml (Fig. 3B). We conclude that the level of serum is not a significant factor when producing HCoV-OC43 and that growing cells in SFM-Optiprep may be advantageous in conditions when one wishes to limit contamination, for example, by exogenous proteins, antibodies or exosomes.

### Sonication may be helpful

**Figure 4:**
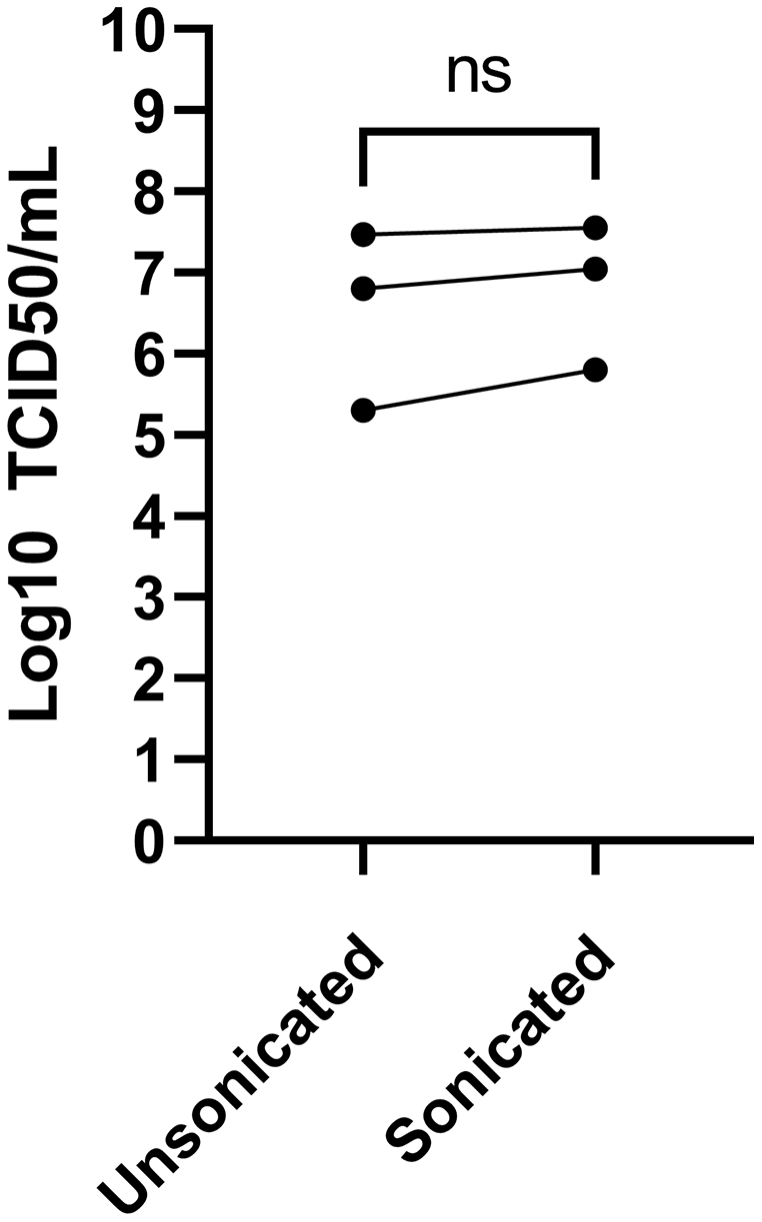
Effect of sonication of viral titers. The media was harvested at 3 dpi from MRC-5 infected cells and were split into two and one of the samples sonicated while the other was kept on ice (see Materials and Methods). Viral titers were then determined by the TCID_50_-IPA method. Three independent experiments are shown individually in the figure (the line indicates the paired samples). Statistical analyses were done by a paired Student t-test.

Sonication is often used to dissociate viral particle aggregates to obtain optimal viral stocks. Sonication of virions released into the tissue culture supernatant was therefore explored using a sonicator equipped with a micro cup horn that we routinely use for preparation of other viruses (Lippé, 2020). Note that such gentler conditions are critical as sonication probes that dip into the samples are too powerful and disintegrate viruses. The non-concentrated tissue culture media harvested 3 days post-infection from MRC-5 infected cells were therefore split in half and treated or not by sonication prior to flash freezing the samples and subsequent titration. Albeit the difference between sonicated and non-sonicated samples was not statistically significant, minor improvements averaging 0.25 log were noted in the viral titers upon sonication (Fig. 4). Clearly, mild sonication did not harm the virus and may in fact be beneficial to get mono-dispersed viral preparations.

### The TCID_50_-IPA method is the most sensitive and practical approach to titer HCoV-OC43

**Figure 5:**
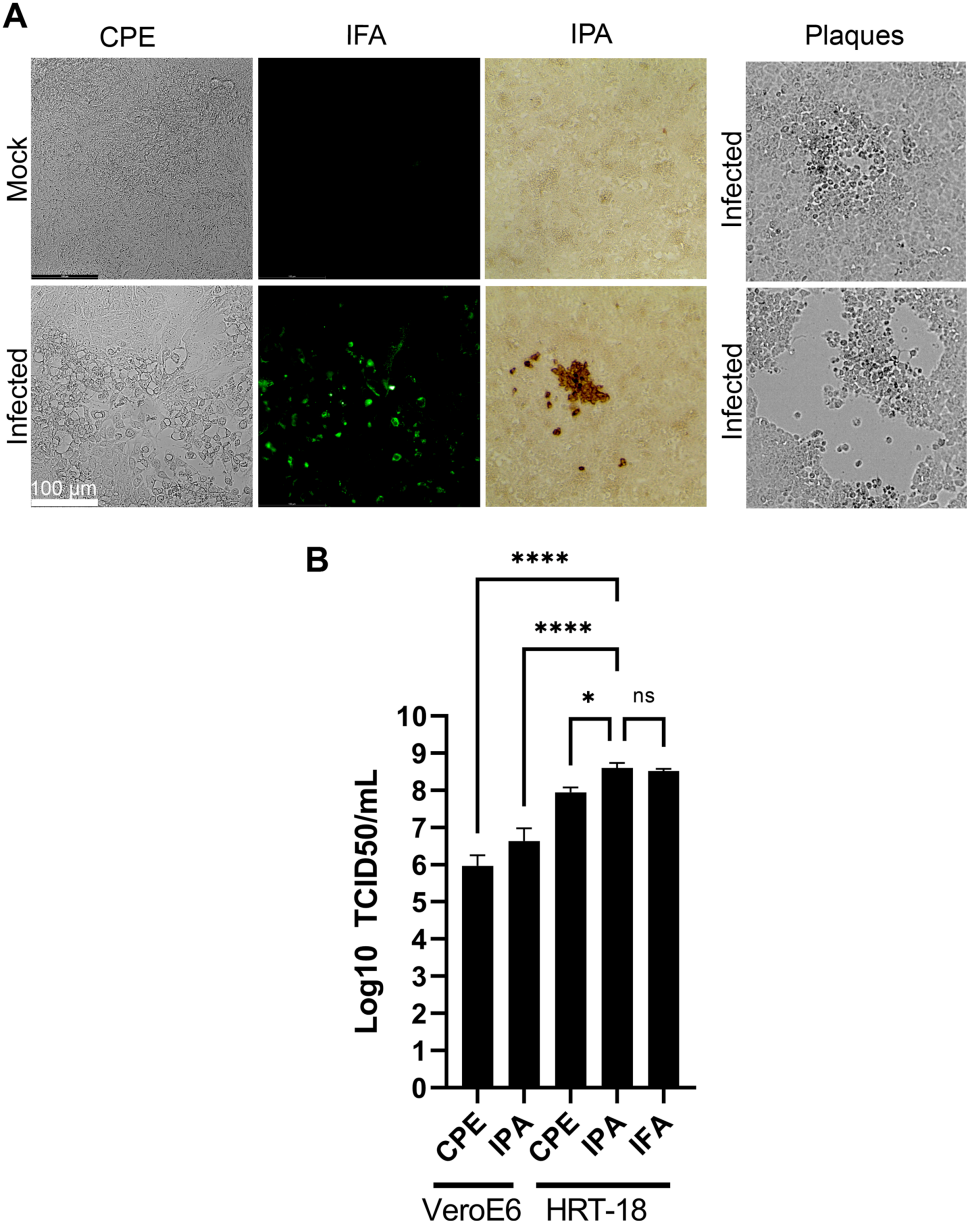
Optimal TCID_50_ method for the titration of HCoV-OC43. A common viral stock produced on MRC-5 cells was used to infect VeroE6 or HRT-18. (A) Typical images of the infected cells by bright field (CPE, IPA) or fluorescence microscopy (IFA). (B) Quantification of the viral yields by TCID_50_-CPE, TCID_50_-IPA and TCID_50_-IFA. Error bars represent SEM (*n* = 3). Statistical analyses were done by one-way ANOVA with Dunnett multiple comparisons (*p < 0.05; ****p < 0.0001).

Various methods have been proposed to quantify coronaviruses, including plaque assays under different semisolid conditions as well as TCID_50_ assays based on CPE or immuno-detection. In our hands, limited CPE was observable under the microscope on the commonly used VeroE6 cells to quantify coronaviruses and plaque assays using various concentrations of agarose or methylcellulose or dyes (crystal violet, neutral red) were not reproducible as they led to either small or poorly defined plaques without signs of infection at their margins making the technique difficult to use (Fig. 5A). This was consistent with several reports where plaques were tiny or irregular in shape with poor contrast (Bracci et al., 2020; Phillpotts, 1983; Reed, 1984; Schirtzinger, Kim & Davis, 2021; Schmidt, Cooney & Kenny, 1979). In contrast, detection of the virus by a TCID_50_ assay based on CPE (TCID_50_-CPE) was feasible because it relies on a plus/minus scoring under the microscope rather than counting plaques (Fig. 5A). Similarly, it was possible to reveal HCoV-OC43 infected cells with primary antibodies targeting the virus and secondary antibodies coupled to horseradish peroxidase and the addition of 3,3’Diaminobenzidine (DAB) (TCID_50_-IPA) or secondary antibodies coupled to a fluorophore (TCID_50_-IFA; Fig. 5A). We therefore sought to compare the efficacy of the three techniques using the same viral stocks. To this end, titrations were performed with the widely used VeroE6 but also the HRT-18 cells, a cell line of choice to monitor HCoV-OC43 (Lambert et al., 2008b). In all cases, manual scoring by individually examining the 96 well plates by microscopy was achieved. This only required a few minutes and was therefore readily amenable to most small to mid-scale experiments. Upon titration on HRT-18 cells, CPE consistently and statistically under evaluated the number of infectious viral particles whereas both immuno-based detection assays were similar (8.9 × 10^7^ TCID_50_/ml for the CPE, 4.0 × 10^8^ TCID_50_/ml for the IPA and 3.3 × 10^8^ TCID_50_/ml for IFA; Fig. 5B). HRT-18 cells also outperformed and proved approximately two logs more sensitive than VeroE6 cells (9.3 × 10^5^ TCID_50_/ml CPE and 4.3 × 10^6^ for IPA). Altogether, this indicated that TCID_50_-IPA or IFA assays on HRT-18 cells represent the most sensitive mean to detect the virus.

### Variation in tropism among HCoV-OC43 isolates

**Figure 6:**
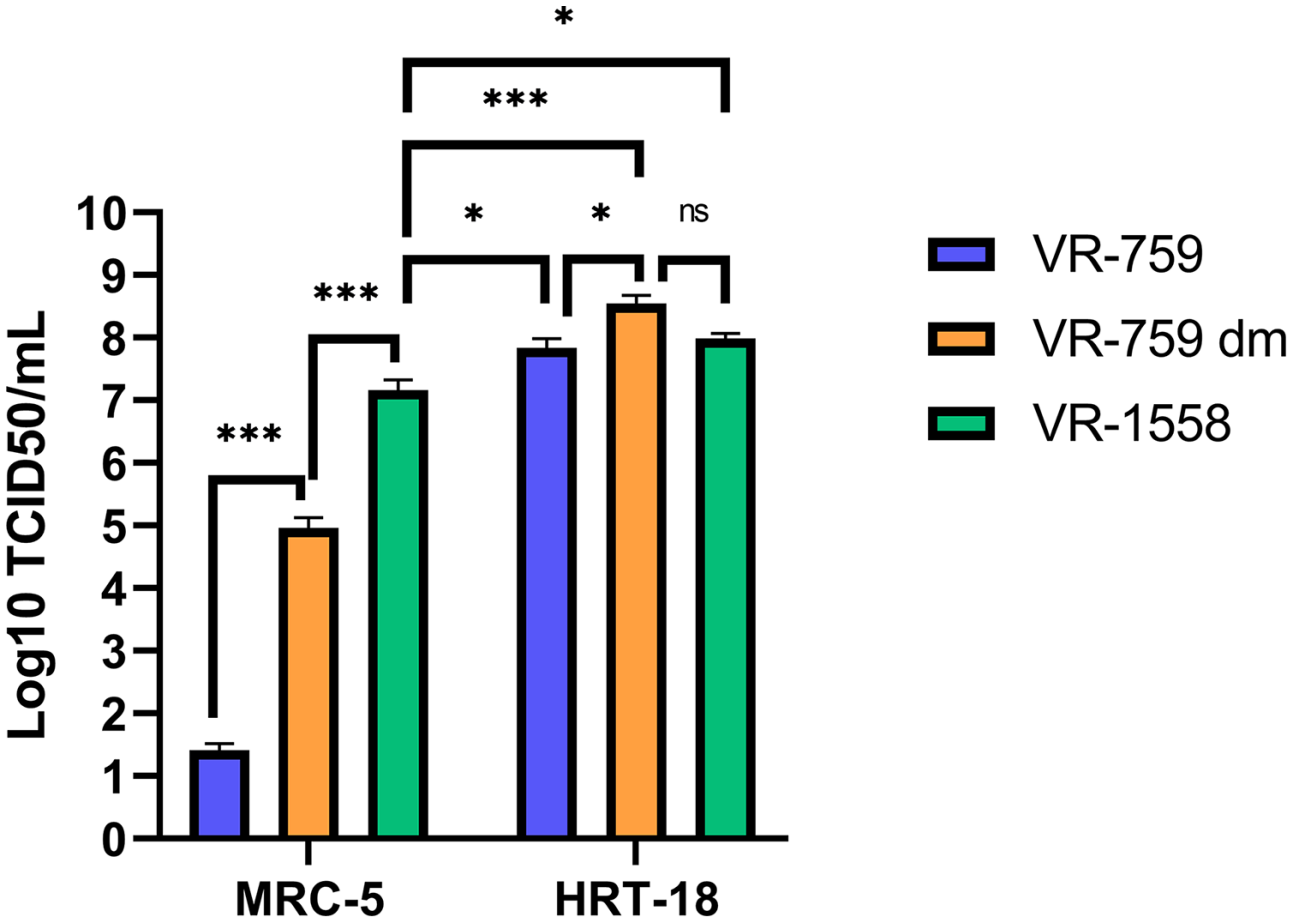
The VR-1558 HCoV-OC43 variant has an increased tropism. MRC-5 and HRT-18 cells were grown to 80% confluence on 6-well plates then infected at an MOI of 0.7 with three HCoV-OC43 variants (VR-759, the HCoV-OC43 rOC/US183-2 double mutant (VR-759 dm) and VR-1558). Three days later, the media was harvested and titered by the TCID_50_-IPA method on HRT-18 cells. Error bars represent SEM (*n* = 3). Statistical analyses were done by one-way ANOVA with Dunnett multiple comparisons (*p < 0.05, ***p < 0.001).

The VR-1558 HCoV-OC43 variant is the only ATCC available HCoV-OC43 isolate nowadays. That newer variant was passaged in mice then adapted in tissue culture from a previously available ATCC VR-759 HCoV-OC43 viral isolate, which was originally isolated from infected organ cultures. Furthermore, the HCoV-OC43 rOC/US183-2 double mutant (VR-759 dm), among other HCoV-OC43 mutants, is derived from the VR-759 variant and contains two mutations within its spike protein that increases its virulence (Favreau et al., 2009). To see if any of these HCoV-OC43 isolates were more suitable, MRC-5 or HRT-18 cells were infected and the virus released into the media titered on HRT-18 cells. In this set of experiments, all three HCoV-OC43 isolates propagated similarly on HRT-18 cells (VR-759: 6.9 × 10^7^; VR-759 double mutant: 3.5 × 10^8^; V-1558: 9.8 × 10^7^ TCID_50_/ml) (Fig. 6). These titers were slightly and significantly reduced when MRC-5 cells were used to grow the VR-1558 isolate (1.5 × 10^7^ TCID_50_/ml) so HRT-18 cells are yet a better cell line to propagate HCoV-OC43. Oddly, the VR-759 isolate very poorly propagated on MRC-5 cells or at all as the observed titer of 2.6 × 10^1^ TCID_50_/ml may well be the input virus, while the VR-759 double mutant displayed an intermediate titer (9.2 × 10^4^ TCID_50_/ml). To delineate differences between these viral isolates, the VR-759 and VR-1558 viral genomes were retrieved from NCBI (NC_006213.1) and ATCC respectively and aligned. As expected, this revealed their close relationship with 99.76% nucleic acid identity with 42 different mutations, including 34 non-silent mutations and three indels (Table 1). Interestingly, VR-1558 harbored the double mutations present in the S protein of the VR-759 double mutant, along with several mutations in the spike and replicase protein, which presumably contribute to the wider tropism of VR-1558. Thus, the current VR-1558 ATCC isolate may be a good choice to grow high titer stocks in a broader range of cell lines, while the VR-759 isolate may be more appropriate to study host restrictions.

**Table 1:** HCoV-OC43 variants. Sequences obtained from NCBI (VR-759 NC_006213.1) and ATCC (VR-1558) were aligned pairwise with BLAST. Forty-two mutations were found, including the 34 non-synonymous mutations or indel shown below. The numbers refer to the VR-759 amino acid sequences. The mutations in the VR-739 double mutant are from the reference below the table.

Gene product	VR-759 dm*	VR-1558
Replicase Polyprotein		317 Arg -> Cys
Replicase Polyprotein		344 Ala -> Val
Replicase Polyprotein		426 Met -> Ile
Replicase Polyprotein		572 Phe -> Ser
Replicase Polyprotein		813 Ala -> Asp
Replicase Polyprotein		1684 His ->Tyr
Replicase Polyprotein		2488 Ser -> Phe
Replicase Polyprotein		2926 Asn -> Ser
Replicase Polyprotein		3782 Arg -> Ser
Replicase Polyprotein		5544 Val -> Glu
Replicase Polyprotein		6059 Met -> Leu
NS2a protein		214 Val -> Leu
HE protein		382 Leu -> Ser
HE protein		383 Pro -> Ser
S protein		24insLeu-Val
S protein		148 Thr -> Ile
S protein	183 His -> Arg	183 His -> Arg
S protein	241 Tyr -> His	241 Tyr -> His
S protein		260insVal-Lys-Asp-Gly
S protein		261 L -> Phe
S protein		365 Ser -> Gly
S protein		388 Gly -> Ala
S protein		533insLys-Ala
S protein		754 Arg -> Ile
S protein		802 Val -> Glu
S protein		869 Leu -> Cys
S protein		899insVal-Asn-Ala-Tyr
S protein		974 Trp -> Leu
S protein		979 Gly -> Arg
S protein		1110 Met -> Ile
S protein		1213 Val -> Gly
N protein		33 Phe -> Val

**Note:**

* Favreau, D. J. et al. 2009 *Virology, 395*(2), 255-267.

## Discussion

Unlike more pathogenic coronaviruses, HCoV-OC43 has been around for several decades but less studied owing to its milder medical footprint. Given its phylogenic link with SARS-CoV-2, it nonetheless constitutes an interesting model to study the basic biology of coronaviruses. Unfortunately, various laboratories propose different protocols to expand and titer HCoV-OC43. The present study addressed this point by comparing four different cell lines (MRC-5, Huh7.5, Vero, HCT-8) and three distinct conditions (temperature, serum, sonication) to produce the virus and by probing the impact of sonication once the virus is harvested. We found that MRC-5, a relevant cell type derived from the lung, is among the best cells to produce the virus. Subsequent testing indicated that HRT-18 cells proved even better than MRC-5 to propagate all tested HCoV-OC43 variants but their colorectal origin makes them a slightly less attractive model. Interestingly, work by Schirtzinger, Kim & Davis (2021) showed that MRC-5 produced high-quality stocks (ratio of 1.2:1 defective to infectious particles) compared to HRT-18 stocks (102:1) at 3 dpi (Schirtzinger, Kim & Davis, 2021), making the former the optimal cell line to make viral stocks. Nonetheless, as MRC-5 cells are rather slow growing, HRT-18 cells may be a useful alternative to rapidly produce viral stocks to subsequently infect relevant cells, with possible caveats for some applications. While sonication of viral stocks did not have any major impact on viral yields, a small increase in titers was noted. As mild sonication (cup-horn) is often used to dissociate viral particle aggregates, this is not surprising and recommendable, but more aggressive sonication (tip) should be avoided as it can denature proteins and break up viral particles.

Our data confirmed previous observations that HCoV-OC43 grows better at 33 °C than 37 °C, presumably because the virus adapted to the lower temperature of the upper respiratory airways. In tissue culture, that lower temperature may also confer an advantage as the slow replication rate of coronaviruses allows healthy uninfected cells to outgrow infected cells at 37 °C (our own observations). In contrast, we found no evidence that serum played any significant role in the propagation of the virus, testing concentrations up to 10%, which is used to culture most cell lines. This was somewhat unexpected given the impact of serum concentration on cellular metabolism and division rate. However, while lower serum concentrations may partially impede cell growth, this could benefit HCoV-OC43 in a similar way as the lower temperature by slowing down the cells. Moreover, our findings are consistent with the literature that suggests a broad range of serum concentrations from 1% to up to 8% to grow HCoV-OC43 (Bracci et al., 2020; Hirose et al., 2021; Lambert et al., 2008c). In fact, viral spread in cells grown in serum-free conditions (EMEM without added serum or commercial serum-free formulation Optipro) proved to be equally efficient at supporting viral propagation. It should be noted that all cell lines look perfectly fine for a few passages in those milieu and we even successfully adapted VeroE6 to Optipro. Of interest, this commercial formulation is spiked with recombinant insulin and epithelial growth factor according to the manufacturer. On the other hand, it is interesting that serum deprivation normally induces autophagy, but coronaviruses seemingly benefit from that pathway (Min et al., 2020; Shroff & Nazarko, 2021). Hence, serum-free conditions may be quite useful for some studies, for example, those that take into consideration the impact of extracellular vesicles that are abundant in serum. Overall, this suggests that MRC-5 and HRT-18 are good models to grow the virus at 33 °C and that reduced serum concentrations could be more cost-effective.

An essential factor in generating viral stocks is the availability of sensitive means to quantify them. To address this, four titration methods were considered, namely plaque assays, TCID_50_-CPE, TCID_50_-IFA and TCID_50_-IPA. Although plaque assays are the gold standard for lytic viruses, they proved unreliable for HCoV-OC43 as poorly defined plaques were only occasionally detected with poor correlations along serial dilutions of the virus. At issue is the CPE limited to a few cells, leading to tiny plaques that are poorly visible despite staining them or large ones that do not show evidence of infection at their edges. As mentioned above, the ability of non-infected cells to outgrow infected ones during viral propagation is problematic. For instance, growing the virus up to 10 days did not lead to increased plaque size (our own observations). These findings corroborated the fact that many studies rely on TCID_50_ assays to monitor coronaviruses, including SARS-CoV-2. One possible alternative around poorly defined plaques is to score CPE positive wells under the microscope to estimate TCID_50_ titers. However, we found that CPE significantly underestimated viral titers compared to IFA and IPA antibody-based detection assays. Since it is important to rely on sensitive assays to properly quantify the virus, IFA and IPA were clearly better than CPE. While the present study relied on a non-commercial antibody generously provided by the Talbot laboratory (Favreau et al., 2009), others have shown that commercial HCoV-OC43 antibodies (*e.g.*, Anti-OC43 N nucleoprotein clone 5427D from Milipore Sigma; Milipore Sigma, Burlington, MA, USA) also perform very well (Schirtzinger, Kim & Davis, 2021) making such approach available to all. The IPA and IFA antibody-based assays were similar in yields to one another but we found the IPA assay easier to use because a simple tissue culture microscope is sufficient and avoids delays and costs associated with fluorescence microscopy. Revealing with DAB is also cheaper than fluorescently tagged secondary antibodies and thus IPA is a more convenient assay that IFA. Finally, the currently available ATCC VR-1558 HCoV-OC43 viral variant clearly has a wider tropism than the original VR-759 variant, presumably owing to the presence of several mutations including the VR-759 double mutations in the spike protein. As with most viruses, the repeated passage of the virus in tissue culture is likely responsible for this change and may even change their preferential route of entry, as for the HCoV-229E coronavirus (Shirato, Kawase & Matsuyama, 2018). Consequently, choosing the right viral variant may be critical depending on the purpose of the studies.

## Supplemental Information

10.7717/peerj.13721/supp-1 Supplemental Information 1 All raw data.

## Additional Information and Declarations

### Data Availability

The following information was supplied regarding data availability: The raw data is available in the Supplemental Files.
